# Drug Use on Mont Blanc: A Study Using Automated Urine Collection

**DOI:** 10.1371/journal.pone.0156786

**Published:** 2016-06-02

**Authors:** Paul Robach, Gilles Trebes, Françoise Lasne, Corinne Buisson, Nathalie Méchin, Monica Mazzarino, Xavier de la Torre, Matthieu Roustit, Patricia Kérivel, Francesco Botré, Pierre Bouzat

**Affiliations:** 1 Ecole Nationale des Sports de Montagne, site de l’Ecole Nationale de Ski et d’alpinisme, Chamonix, France; 2 HP2, Université Grenoble Alpes, Grenoble, France; 3 HP2, INSERM, Grenoble, France; 4 Pôle Urgences Médecine Aigüe, CHU de Grenoble, Grenoble, France; 5 Département des Analyses, Agence Française de Lutte contre le Dopage, Chatenay-Malabry, France; 6 Laboratorio Antidoping, Federazione Medico Sportiva Italiana, Rome, Italy; 7 Pôle Recherche, Pharmacologie Clinique INSERM CIC1406, CHU de Grenoble, Grenoble, France; 8 Dipartimento di Medicina Sperimentale, « Sapienza » Università di Roma, Rome, Italy; 9 Pôle Anesthésie-Réanimation, CHU de Grenoble, Grenoble, France; 10 Institut des Neurosciences, INSERM U836, Université Grenoble Alpes, Grenoble, France; Medical University of Graz, AUSTRIA

## Abstract

Mont Blanc, the summit of Western Europe, is a popular but demanding high-altitude ascent. Drug use is thought to be widespread among climbers attempting this summit, not only to prevent altitude illnesses, but also to boost physical and/or psychological capacities. This practice may be unsafe in this remote alpine environment. However, robust data on medication during the ascent of Mont Blanc are lacking. Individual urine samples from male climbers using urinals in mountain refuges on access routes to Mont Blanc (Goûter and Cosmiques mountain huts) were blindly and anonymously collected using a hidden automatic sampler. Urine samples were screened for a wide range of drugs, including diuretics, glucocorticoids, stimulants, hypnotics and phosphodiesterase 5 (PDE-5) inhibitors. Out of 430 samples analyzed from both huts, 35.8% contained at least one drug. Diuretics (22.7%) and hypnotics (12.9%) were the most frequently detected drugs, while glucocorticoids (3.5%) and stimulants (3.1%) were less commonly detected. None of the samples contained PDE-5 inhibitors. Two substances were predominant: the diuretic acetazolamide (20.6%) and the hypnotic zolpidem (8.4%). Thirty three samples were found positive for at least two substances, the most frequent combination being acetazolamide and a hypnotic (2.1%). Based on a novel sampling technique, we demonstrate that about one third of the urine samples collected from a random sample of male climbers contained one or several drugs, suggesting frequent drug use amongst climbers ascending Mont Blanc. Our data suggest that medication primarily aims at mitigating the symptoms of altitude illnesses, rather than enhancing performance. In this hazardous environment, the relatively high prevalence of hypnotics must be highlighted, since these molecules may alter vigilance.

## Introduction

Mont Blanc (4810m altitude), at the border between France and Italy, is the highest mountain in Western Europe and one of the most climbed summits in the world. About 35,000 people attempt to reach the summit every year [1]. Despite its popularity, an ascent of Mont Blanc is considered to be a highly demanding exercise requiring good aerobic performance, technical expertise and altitude acclimatization. However anecdotal evidence indicate that many people attempting the summit do not have the experience, physical condition or skill-set required, and/or are not sufficiently well-acclimatized [2]. This may explain why exhaustion is commonly reported among Mont Blanc climbers.

To avoid altitude related illnesses and/or exhaustion, and ultimately to increase their chance of reaching the summit, climbers may use medications, with or without prescription. Several categories of drugs may be relevant to this purpose: first, since the rapid altitude gain increases the risk of acute mountain sickness, prophylactic treatments with acetazolamide [3] or glucocorticoids [4] may be considered; second, the potential risk of exhaustion may also incite some climbers to take stimulants [5]; third, the performance-enhancing effect of phosphodiesterase 5 (PDE-5) inhibitors at high altitude may prompt people to use these drugs to optimize their ascent [6]; and fourth, high-altitude sleep disturbances that may otherwise compromise the summit push can be alleviated by specific hypnotic drugs [7, 8].

It may be worthwhile to highlight that, with the exception of hypnotics, all the drugs mentioned above possess a favorable effect at altitude [6, 9, 10]; acetazolamide, glucocorticoids and stimulants are indeed banned in sports, being included in the list of prohibited substances of the World Anti-Doping Agency (WADA) [11]. As alpinism is not subject to anti-doping rules, any objections to the use of medications are based on ethical or safety concerns. Indeed, although prophylactic medication against altitude sickness is justified in some cases [12], drug use is always associated with some risks. First, all the above mentioned drugs have side effects whose consequences can become highly problematic in a remote alpine environment: acetazolamide increases urine frequency that may exaggerate dehydration; short-term treatment with glucocorticoids may induce hyperglycemia [13]; stimulants may lead to cardiovascular complications, hypertension, and/or thermoregulatory problems [14]; PDE-5 inhibitors are commonly associated with headache and rarely with visual disturbances; finally, residual effects of hypnotics may alter psychomotor and cognitive functioning [15]. Second, if drugs are used to push physical or psychological barriers and to delay the onset of fatigue in order to reach the summit, they may, ultimately, lead to greater levels of exhaustion and/or decompensation during the climb or the descent. Third, in the case they are taken to induce sleep the night before the ascent, they can cause reduced reactivity in potential emergencies.

Except for acetazolamide, which is widely used during high altitude fast climbs [16], there is limited information available on the prevalence of ergogenic drug use amongst mountaineers. One case report [17] and numerous anecdotic reports of drug use [18] suggest that the practice is widespread, notably on iconic summits. Reinhold Messner, the renowned Italian mountaineer, has suggested that up to 90% of those attempting to conquer Mount Everest may use drugs [19]. To the best of our knowledge, drug consumption amongst alpinists attempting to ascend Mont Blanc has never been evaluated based on the direct analysis of their residues/metabolites in body fluids.

The aim of this study was to verify whether the use of performance-enhancing drugs is prevalent amongst alpinists ascending Mont Blanc. To quantify this phenomenon, we developed and conducted an automated, blinded collection of individual urine samples, in the mountain huts located on the two main access routes to the summit. The urine samples were analyzed for a large number of drugs/metabolites, belonging in particular to the classes of diuretics, glucocorticoids, stimulants, hypnotics and PDE-5 inhibitors.

## Methods

### Ethical aspects

As this study was designed to investigate a phenomenon which is generally negatively perceived (performance-enhancing drug use), it is susceptible to a high level of selection bias i.e. people using drugs may want to hide this fact and not be included in the study. In order to reduce this bias, participants were not individually informed about the study and consent was not sought. A notice in French and in English was posted on the main entrance to the bathroom area (serving all toilets and urinals), saying that urine samples might be randomly collected for analysis, without specifying the purpose of the analyses. For ethical reasons, no clinical or demographic information was collected. Furthermore, we took all possible precautions to make the identification of subjects impossible. Study Ethics approval was obtained on January 14^th^ 2013 (CECIC Rhône-Alpes-Auvergne, Clermont-Ferrand, France, IRB: 5891). Formal authorizations to modify one urinal in each of the high-altitude huts were obtained from the *Fédération Française des Clubs Alpins et de Montagne* (Goûter hut) and *Compagnie des Guides de Chamonix* (Cosmiques huts).

### Ascent patterns of Mont Blanc

Two popular routes lead to the top of Mont Blanc. The first one, via Goûter hut and “Arête des Bosses”, starts from the “Nid d’Aigle” train station (2272m). Climbers typically leave the valley in the morning and reach the Goûter hut (3845m) in the afternoon. They summit Mont Blanc the next morning and return in the valley in the afternoon. Total time spent above 2500m is around 30 hours. The second one, via Cosmiques hut and “Trois Mont Blanc”, starts from “Aiguille du Midi” cable car station (3842m). Climbers typically leave the valley in the afternoon and reach the Cosmiques hut (3615m) following a one-hour climb. They attain Mont Blanc the next morning and return in the valley in the afternoon. Total time spent above 2500m is around 24 hours.

### Automatic collection of urine samples

**Fig 1:**
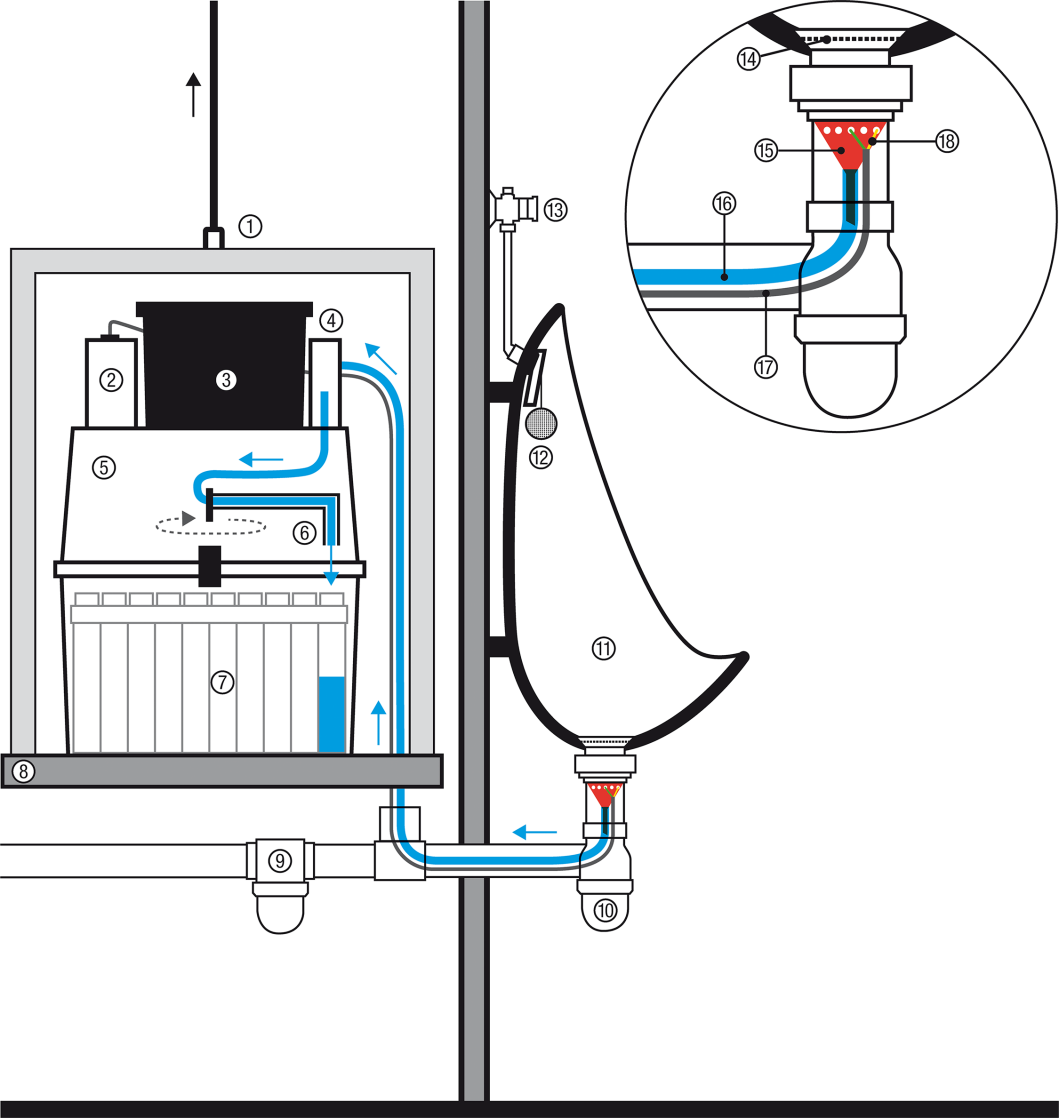
Diagram of the automatic urine collection system. ① mobile soundproof box with a pulley block; ② battery; ③ controller, motor and software; ④ roller pump; ⑤ sampler’s hood; ⑥ rotating sampler arm; ⑦ multi-container cassette (24 × 500 ml); ⑧ fixed soundproof support base; ⑨ siphon passage; ⑩ modified siphon; ⑪ urinal; ⑫ basket containing salt tablets; ⑬ manual flush; ⑭ filter grid; ⑮ funnel sieve (with holes); ⑯ sampling tube; ⑰ waterproof electric wire; ⑱ liquid presence detector, composed of two isolated wires entering the funnel through distinct holes. Bare wire ends (1 cm) are positioned horizontally inside the funnel so as not to touch each other. It should be noted that with the present system, cross-contamination of urine may occur between successive samples (see Methods). A possible improvement of the system, minimizing residual liquid volume and therefore potential contamination, would consist in positioning the tip of the sampling tube (⑯) within the funnel (⑮) while maintaining the collector tube vertical throughout its course, by means of a side hole in the upper part of the drainpipe. This change could not be implemented during the course of the study due to the complexity of the technical modification in this extreme field environment.

Urine samples were collected from the urinals used by male individuals at the Goûter and the Cosmiques mountain huts. Since our system could not be adapted to toilets where urine is mixed with water systematically, women could not be included in the present analysis. Each individual sample was collected in a separate container in a custom-made invisible and inaudible automatic system (Fig 1). The multi-sample system (24 × 500 ml) operated via an automatic, battery-driven water sampler (portable model 3700C, Teledyne ISCO, Lincoln NE, USA) connected to a liquid presence detector (model LD90) with a detection threshold of 25 ± 10 micro siemens (μS)/cm. The intake suction tubing (one meter) and the detector (modified to reduce its size) were positioned above the syphon on the drainpipe of one of the urinals in each hut (Fig 1). The average volume of urine collected in each sample was 67 ml. The sampler was acoustically isolated and installed in an adjacent service duct. When the urinal was flushed, a water sample was drawn. In both huts, domestic water obtained by melting ice from the glacier was characterized by very low conductivity (< 25 μS/cm). Thus, to ensure proper water detection by the system, water conductivity was increased with salt tablets (Axal, Esco, Hannover, Germany) positioned in the water inlet circuit. This procedure did not interfere with subsequent drug analyzes. An air purge was initiated before and after each sample.

The sampling procedure took place over 14 days for the Cosmiques hut and 21 days for the Goûter hut between July and September 2013. In each hut, one urinal was fitted with the sampling system. At the Cosmiques hut, which serves Mont Blanc and other peaks, the sampler operated from 00:00 a.m. to 04:00 a.m. corresponding to the time of departures for Mont Blanc. In all, the Cosmiques hut has three urinals and three toilets, all located in the same area accessible 24h per day. At the Goûter hut, which only serves Mont Blanc, the sampler (located at the restaurant level, for technical reasons) operated from 01:30 a.m. to 09:00 p.m. In all, the Goûter hut has five urinals and six toilets, divided into three areas: 1) at the restaurant level (two urinals and three toilets, area closed between 09:00 p.m. and 01:30 a.m.), 2) at the dormitory first floor (two urinals and two toilets, area accessible 24h per day) and 3) at the dormitory second floor (one urinal and one toilet, area accessible 24h per day). Sampling automatically stopped after 24 samples (each sampling time was recorded), then a new clean multi-sample cassette was inserted, the tube rinsed and the sampler reprogrammed for the next night. All operations (programming, cleaning, maintenance, transport) were supervised by members of the research team, with the assistance of a trained staff member in each hut. The full multi-sample cassettes were stored at -20°C in the hut until being transported weekly by helicopter or on foot to the laboratory in Chamonix. The experimental urinals were not used by the hut staff.

Since our sampling process involved a residual volume of liquid in the tube (up to 4 ml), contamination of the next sample could not be excluded. The rate of potential cross-sample contamination was assessed during a separate on-site experiment, using urine samples titrated with selected drugs. The rate of contamination between two successive urine samples (the first titrated, the second blank) was 4.7% for betamethasone, 0.0% for prednisolone, 6.0% for tetrahydrocannabinol, 5.7% for acetazolamide and 12.8% for hydrochlorothiazide. These values correspond to averages from two separate tests (urinary concentrations were measured in duplicate for each test).

### Analytical procedures

The urine samples were screened for diuretics, glucocorticoids and stimulants by the French WADA-accredited laboratory (Département des Analyses, Agence Française de Lutte contre le Dopage). Confirmatory analyses were performed for diuretics and some stimulants. A wider screening by the same lab included other substances from the WADA 2016 list of prohibited substances [11]: anabolic agents, beta-2 agonists, metabolic modulators, narcotics, cannabinoids and beta-blockers. PDE-5 inhibitors, benzodiazepines and related substances were analyzed (screening and confirmation) by the Italian WADA-accredited laboratory (Laboratorio Antidoping, Federazione Medico Sportiva Italiana) following a specific analytical procedure [20]. The list of the searched substances and the details of the analytical procedures are available in S1 File.

### Data expression

Each substance was considered independently in the analysis. The status for each substance in a urine sample could be negative, positive or contaminated. The latter was applied when the concentration was less than or equal to that measured in the previous sample. We chose this conservative approach to avoid any risk of including false positive results in our analysis. The proportion of positive cases (in %) for a given substance was calculated as follows: number of positive samples for this substance ÷ (number of samples analyzed in total–number of samples contaminated by this substance) × 100.

We also calculated the rate of positive samples, whatever the type of substance. The status of the whole sample could be positive if at least one substance was found positive in this sample, regardless of other substances possibly contaminating the sample; negative if no substance could be detected in this sample and no substance contaminated this sample; contaminated if no substance was found positive in this sample and at least one substance considered as contamination was found.

### Data analysis

Data analysis was descriptive. Quantitative variables are expressed as proportions and a 95% confidence interval (95% CI). Continuous variables are expressed as means and standard deviations (SD). The precision of the estimation was calculated *a priori*. For a sample size of 400, a two-sided 95% confidence interval for a single proportion using the large sample normal approximation extends <0.05 from the observed proportion, for an expected proportion of 0.4 or lower (nQuery Advisor® v7, Statistical Solutions Ltd, Cork, Ireland).

## Results

Considering all 430 urine samples analyzed, the proportion of positive samples in both huts was 35.8% (31.3–40.3%), that is 31.4% (24.1–38.7%) and 38.3% (32.5–44.1%) in the Cosmiques and the Goûter huts, respectively. The proportion of negative samples was 48.8% (44.1–53.5%) and contamination occurred in 15.3% (11.9–18.7%) of the samples. The classes of substances most often detected in urine samples were diuretics and hypnotics (Table 1). Glucocorticoids and stimulants were identified in urine samples to a lesser extent. No PDE-5 inhibitors were detected in the urine samples. The wider screening found some other substances in the samples, such as cannabinoids, narcotics and beta-blockers. We detected neither anabolic agents, nor beta-2 agonists.

**Table 1:** Prevalence and concentrations of drugs found in urine from Mont Blanc climbers.

	Positive samples, % (95% CI)			Concentrations, ng/ml
Substance	Goûter hut	Cosmiques hut	Both huts	Both huts
				Mean ± SD (min-max)
**Diuretics**	24.9 (19.3–30.4)	19.1 (12.7–25.6)	22.7 (18.5–27.0)	
Acetazolamide	22.4 (17.1–27.7)	17.6 (11.3–23.9)	20.6 (16.5–24.7)	44,630 ± 96,857 (20–491,100)*
Hydrochlorothiazide	1.9 (0.2–3.5)	1.3 (0.0–3.1)	1.6 (0.4–2.9)	1,935 ± 3,620 (4–10,048)*
**Hypnotics**	12.7 (8.4–17)	13.3 (7.6–19.1)	12.9 (9.5–16.4)	
Zolpidem	8.2 (4.7–11.7)	8.7 (4.0–13.4)	8.4 (5.6–11.2)	9 ± 23 (0.1–127)*
Oxazepam	1.7 (0.0–3.4)	1.4 (0.0–3.3)	1.6 (0.3–2.8)	67 ± 85 (3–222)*
Zopiclone	1.3 (0.0–2.7)	1.4 (0.0–3.3)	1.3 (0.2–2.4)	185 ± 313 (11–739)*
Lorazepam	0.9 (0.0–2.2)	0.0	0.5 (0.0–1.3)	2,858 ± 3,816 (160–5,556)*
Bromazepam	0.4	0.0	0.3	23* ^(n = 1)^
Zaleplon	0.0	0.7	0.3	3* ^(n = 1)^
Brotizolam	0.0	0.7	0.3	1* ^(n = 1)^
**Glucocorticoids**	3.7 (1.5–6.0)	3.3 (0.5–6.1)	3.5 (1.8–5.3)	
Prednisone	2.2 (0.5–4.0)	1.3 (0.0–3.1)	1.9 (0.6–3.2)	391 ± 306 (11–776)
Prednisolone^§^	1.9 (0.2–3.5)	1.3 (0.0–3.1)	1.6 (0.4–2.9)	939 ± 1,346 (11–3,823)
Betamethasone	1.1 (0.0–2.3)	0.0	0.7 (0.0–1.5)	25 ± 15 (13–42)
Budesonide	0.4 (0.0–1.1)	0.6 (0.0–1.9)	0.5 (0.0–1.1)	15 ± 6 (11–19)
Methylprednisolone	0.0	1.3 (0.0–3.1)	0.5 (0.0–1.1)	639 ± 831 (51–1,226)
**Stimulants**	4.1 (1.7–6.5)	1.3 (0.0–3.1)	3.1 (1.4–4.7)	
Caffeine	1.1 (0.0–2.3)	0.6 (0.0–1.9)	0.9 (0.0–1.8)	7,725 ± 629 (7,000–8,300)
Benzoylecgonine^‡^	1.1 (0.0–2.3)	0.0	0.7 (0.0–1.5)	253 ± 263 (68–439)*
Pseudoephedrine	0.7 (0.0–1.7)	0.0	0.5 (0.0–1.1)	10,940 ± 14,228 (879–21,000)
Ephedrine	0.4	0.0	0.2	9 ^(n = 1)^
N-ethylnicotinamide	0.4	0.0	0.2	299 ^(n = 1)^
Heptaminol	0.0	0.6	0.2	127* ^(n = 1)^
Dihydrobupropion	0.4	0.0	0.2	665 ^(n = 1)^
**Cannabinoids**				
THC	4.6 (2.0–7.1)	2.6 (0.1–5.1)	3.8 (2.0–5.7)	44 ± 44 (8–152)*
**Narcotics**	2.2 (0.5–4.0)	3.3 (0.5–6.1)	2.6 (1.1–4.1)	
Codeine	1.5 (0.0–2.9)	2.6 (0.1–5.1)	1.9 (0.6–3.2)	379 ± 490 (11–1,297)
Morphine^§^	1.5 (0.0–2.9)	2.6 (0.1–5.2)	1.9 (0.6–3.2)	70 ± 91 (6–274)
Methadone	0.4	0.0	0.2	5* ^(n = 1)^
Hydrocodone	0.4	0.0	0.2	480 ^(n = 1)^
Tramadol	0.0	0.6	0.2	358 ^(n = 1)^
**Beta-blockers**	1.5 (0.0–2.9)	0.6 (0.0–1.9)	1.2 (0.1–2.2)	
Betaxolol	0.7 (0.0–1.7)	0.0	0.5 (0.0–1.1)	201 ± 16 (190–212)*
Metoprolol	0.7 (0.0–1.7)	0.0	0.5 (0.0–1.1)	44 ± 14 (34–54)*
Metoprolol acid^§^	0.7 (0.0–1.7)	0.0	0.5 (0.0–1.1)	109 ± 129 (17–200)
Bisoprolol	0.0	0.6	0.2	64* ^(n = 1)^
**Metabolic modulators**	0.7 (0.0–1.7)	0.0	0.5 (0.0–1.1)	
Methoxytamoxifen	0.4	0.0	0.2	25 ^(n = 1)^
Anastrozole	0.4	0.0	0.2	250 ^(n = 1)^

Data are derived from samples collected in the Goûter (n = 274) and the Cosmiques huts (n = 156). CI, confidence interval; THC, tetrahydrocannabinol

^§^indicates that this substance was always detected concomitantly with the previous one, within the same urine sample. Only the first substance (i.e. prednisone, codeine or metoprolol) was recorded for positive cases.

^‡^benzoylecgonine is the main cocaine metabolite.

*values are derived from confirmatory analyses. Individual data of drug concentrations in positive urine samples are available in S1 Table.

In both huts, the main substances detected in urine samples were acetazolamide and zolpidem (Table 1). Prednisone was the most frequently detected glucocorticoid, while caffeine and the cocaine metabolite benzoylecgonine were the most often found stimulants. Oxazepam was the most frequently detected anxiolytic hypnotic drug. Prevalence data were generally similar between the two huts. Table 1 also reports the urine mean concentrations of the different substances.

Drug combinations are presented in Table 2. In total, thirty-three urine samples were found positive for at least two substances, the most frequent combinations being the association of acetazolamide with a hypnotic (2.1%) followed by that of acetazolamide with a glucocorticoid (1.9%). The combination of two diuretics (acetazolamide and hydrochlorothiazide) was seen in 0.9% of the samples.

**Table 2:** Combinations of drugs found in urine from Mont Blanc climbers.

Substance 1	Substance 2	Substance 3	Goûter hut	Cosmiques hut	Bothhuts
			Number of cases		
Acetazolamide	Zolpidem		5	2	7
Acetazolamide	Hydrochlorothiazide		2	2	4
Acetazolamide	Prednisone		3	1	4
Acetazolamide	Betamethasone		3	0	3
Acetazolamide	Methylprednisone		0	1	1
Acetazolamide	Metoprolol		1	0	1
Acetazolamide	Zopiclone		0	1	1
Acetazolamide	Zolpidem	Bisoprolol	0	1	1
Acetazolamide	Benzoylecgonine^‡^	Pseudoephedrine	1	0	1
Acetazolamide	Codeine	Hydrocodone	1	0	1
Zolpidem	Caffeine		1	0	1
Zolpidem	Prednisone		1	0	1
Zolpidem	THC		0	1	1
Zolpidem	Zaleplon		0	1	1
Zolpidem	Codeine		0	1	1
Zolpidem	Dihydrobupropion		1	0	1
Prednisone	THC		0	1	1
Hydrochlorothiazide	Prednisone	Benzoylecgonine	1	0	1
**Total**			20	12	32

THC, tetrahydrocannabinol. In addition to the thirty-two cases of drug combinations with two or three substances, one urine sample collected at the Goûter hut contained five substances: methoxytamoxifen, anastrozole, caffeine, lorazepam and methadone (not shown in the table).

^‡^benzoylecgonine is the main cocaine metabolite.

## Discussion

To our knowledge, this is the first study examining the prevalence of drug use based on blinded, random urine sample collection. The selection of the target analytes was based on the following criteria: i) enhancement of performance (based on the current anti-doping rules for competitive sports); ii) therapeutical/preventive pharmacological treatments, and iii) safety issues.

At least one drug was detected in about one in three urine samples collected from a random sample of male individuals climbing the Mont Blanc. Two molecules were prevalent among this set of urine samples: the diuretic acetazolamide and the hypnotic zolpidem. In contrast, the prevalence of other substances potentially increasing physical performance at altitude, i.e. glucocorticoids and stimulants, was low, and no PDE-5 inhibitors were detected. Finally, our results indicate that thirty-three urine samples contained more than one drug, suggesting that some people took multiple medications for the ascent of Mont Blanc.

With regard to pharmacological treatment of acute mountain sickness, the percentage of urine samples containing acetazolamide appears to be high: our data on Mont Blanc confirms those reported in a previous survey on Mount Kilimanjaro, showing that 33% of the climbers were taking acetazolamide [16]. Indeed, history of acute mountain sickness, insufficient acclimatization and/or lack of previous high-altitude experience (and therefore the fear of being susceptible to altitude sickness) are likely reasons for frequent acetazolamide use also on Mont Blanc. Medical guidelines [4] and review articles [12, 21, 22] recommending acetazolamide when a rapid ascent to high altitude cannot be avoided may also encourage people to use this drug on Mont Blanc. Such practice is however questionable since the efficacy of acetazolamide for particularly high and fast climbs such as Mont Blanc (altitude gain of almost 4,000 m within 24-48h) remains uncertain [12]. Although any pharmacokinetic interpretation must be made with great caution in the absence of any information regarding drug intake characteristics, our data suggest “moderate-dose” treatment among Mont Blanc climbers, since the mean urine concentration of acetazolamide was similar to levels measured 8–24 hours after a single oral dose of 250 mg [23, 24] and 125 mg orally is generally considered “low-dose” treatment. Finally, we cannot exclude that the use of acetazolamide could be over-detected, since climbers using this drug may urinate more frequently and thus be slightly oversampled in the population.

Although difficulty in sleeping is a common problem at high altitude, treatment of altitude sleep disorder with hypnotics is only partially documented and therefore not always advisable, as it should be reserved for those whose sleep problems are very severe [25]. In our study, more than 10% of the urine samples randomly collected from male climbers contained hypnotics, which is approximately twice the proportion observed in a large French epidemiological study [26]. Hypnotic drugs could be harmful to climbers when wake-up occurs shortly after medication intake (typically 4–5 hours, as climbers usually leave the huts 3 to 4 hours before sunrise), suggesting possible residual negative effects on vigilance [15], at a time when a high level of alertness is required. It cannot be excluded that, in this particular setting, even short half-life benzodiazepine-like agents such as zolpidem may diminish cognitive performance.

Contrary to our hypothesis, performance enhancers were not frequently detected: few urine samples were found positive for glucocorticoids (despite prospective trials have established a benefit for the glucocorticoid dexamethasone in the prevention of acute mountain sickness) [4], and PDE-5 inhibitors were totally absent. The presence of stimulants among urine samples of Mont Blanc climbers was also marginal (with the notable exception of three samples containing cocaine, the highest concentration suggesting recent use ≤ 24h) [27], in contrast to previous data indicating amphetamine use among climbers in the Swiss Alps [5]. The presence of tetrahydrocannabinol (THC) in some samples (3.8%) raises concerns about possible impairments of cognitive and psychomotor performance [28], however the long half-life of THC did not specifically indicate current use.

Finally, the detection of combinations of drugs in our set of urine samples raises questions about potential adverse effects related to drug interactions among climbers. In particular, it would be prudent to avoid recommending co-medication with acetazolamide and zolpidem in the absence of sufficient data [25], and the combination of diuretics (acetazolamide and hydrochlorothiazide) may exacerbate dehydration and/or hypokalemia during prolonged strenuous exercise at high altitude. The issue of dangerous effects of drug-drug interactions in this extreme environment becomes more worrying considering that climbers may concomitantly use other classes of drugs not studied here (such as nonsteroidal anti-inflammatory drugs).

We acknowledge several limitations to our blinded urine collection method. First, sampling was limited to male individuals. Although data are not known precisely, the proportion of women climbing Mont Blanc was estimated to be between 10 and 20%. In our male population, the absence of information on subjects (such as demography, mountaineering experience, performance, or medical conditions) prevented us to know why drugs were used, and in particular if the use of prevalent drugs such as acetazolamide or zolpidem could be problematic or on the contrary beneficial. Second, we probably underestimate the prevalence of drug use because of the withdrawal of all possibly contaminated samples from analysis (see Methods). Our approach may appear too conservative considering the relatively low cross-contamination rate (<13%). However, we did not use a cut-off value (e.g. of 15%) to confirm whether a sample was contaminated or not, since the titration experiment was limited to only a small selection of drugs, with fixed concentrations. We propose a possible improvement of the sampling system to minimize potential cross-contamination and therefore missing values (see legend to Fig 1). Third, because strict anonymity was ethically obligatory, we did not monitor the rate of redundant samples (i.e. coming from the same individual). At the Cosmiques hut, where the sampler operated only at night, we speculate that redundancy was low since 1) mountaineers are usually in a hurry to leave the hut (<1 hour between awakening and departure) leading them to urinate only once; and 2) six toilets/urinals were simultaneously accessible. At the Goûter hut (where the sampler operated from 01:30 a.m. to 09:00 p.m.), the fact that people did not use the experimental urinal during sleeping time, and furthermore could always choose between 11 toilets/urinals during the sampling period leads us to speculate that redundancy was also limited. However, some people being creatures of habit, they might use the same urinal each time, thus it is not excluded that our results may have overestimated drug use. Alternatively, assuming that redundancy rate was comparable between people using drugs and those who did not suggests that the overall impact of redundancy on our prevalence data remained low. Fourth, since one urinal was fitted with the sampling system in each hut, climbers who never used this urinal were not included in the study population. Our sample would have been more representative of the climbers’ population if all urinals had been equipped, however such a design would have probably increased the rate of redundant samples. Fifth, as the automated sampling procedure could not distinguish a single dose from regular long-term use, data interpretation must be cautious, especially for prevalent drugs such as hypnotics [29]. However, whatever the therapeutic class, it seems difficult to compare our international climbers with population-based cohorts. Indeed, there is a lack of self-reported prevalence data on drug use in the general population and little data from international studies are readily available. Finally, it would have been interesting to investigate nonsteroidal anti-inflammatory drugs in the present study, as these compounds are commonly used by mountaineers for preventive/prophylactic treatment or acute mountain sickness, and may furthermore enhance performance by reducing the chance of getting sick. However, such analyses were not conducted since these molecules are not on the WADA list of prohibited substances and therefore WADA-accredited laboratories do no implement assays for these drugs.

## Conclusions

We show that about one third of the urine samples collected from a random sample of male climbers contained one or several drugs. This suggests that drug use is frequent among climbers on Mont Blanc. The drugs used seem primarily intended to alleviate symptoms related to altitude sickness, but would not represent a doping behavior. Alternatively, the use of acetazolamide, particularly as a preventive medication, can be viewed as “doping” since it may improve performance at high altitude by decreasing the chance of getting sick. The relatively high prevalence of hypnotics, as well as the use of combined medication (presumably with limited knowledge of potential drug-drug interactions and adverse effects) in this hazardous environment could affect climbers’ safety.

A future perspective is to extend this novel sampling method to other contexts, where information is needed on the prevalence of the use of drugs while limiting selection bias. Examples might include research on the prevalence on doping among athletes participating in popular sporting events (such as endurance competitions, where urinals are generally available on site) which may be relevant for anti-doping policies; studies on psychotropic substance use among drivers (by using gas station bathrooms) for the purposes of road safety; and prevalence of drug abuse among participants to social mass meetings, e.g. popular music festivals. It may be worth asking why similar studies have not been previously implemented during popular sporting events. Ethical aspects might be one reason. Even in our study, which involved a sport not subject to anti-doping rules, respecting strict anonymity was a key point requested by the ethics committee. It can be therefore speculated that if such a program should be implemented in a competitive sport, investigators should take all necessary measures to ensure that their data cannot be used for other purposes than science (e.g. doping control).

## Supporting Information

S1 File Description of Analytical Procedures. (DOC)

S1 Table Individual data of drug concentrations in urine samples from Mont Blanc climbers. (DOCX)
