# Supplementary material for: Drug Use on Mont Blanc: A Study Using Automated Urine Collection
Source: PLoS One. 2016 Jun 2;11(6):e0156786. doi: 10.1371/journal.pone.0156786 (PMC4890938; doi:10.1371/journal.pone.0156786)
Supplement: S1 File — (DOC) [file pone.0156786.s001.doc]

**S1 File. Description of Analytical Procedures.**

**Table of contents**

1. Analytical procedures for drug analyses (except benzodiazepines and phosphodiesterase 5 inhibitors) p. 2
   1. Screening procedure by liquid chromatography - tandem mass spectrometry (LC MS/MS) with enzymatic hydrolysis p. 2
   2. Screening procedure by LC-MS/MS without enzymatic hydrolysis p. 3
   3. Screening procedure for anabolic steroids and metabolic modulators by gas chromatography (GC)-MS/MS p. 3
   4. Confirmatory analyses p. 4
2. Analytical procedures for benzodiazepines (and related substances) and PDE-5 inhibitors p. 5
3. Supplementary tables p. 6

S2 Table. Mass spectrometric conditions (screening procedure with enzymatic hydrolysis) p. 7

S3 Table. Mass spectrometric conditions (screening procedure without enzymatic hydrolysis) p. 8

S4 Table. Confirmatory analyses p. 9

S5 Table. Mass spectrometric conditions for benzodiazepines and PDE-5 inhibitors p. 10

1. References p. 11
2. **Analytical procedures for drug analyses (except benzodiazepines and phosphodiesterase 5 inhibitors)**
   1. **Screening procedure by liquid chromatography - tandem mass spectrometry (LC-MS/MS) with enzymatic hydrolysis**

*Sample pre-treatment*

4 mL of urine was incubated with 1 mL of phosphate buffer 0.2 M (pH 6.5), 50 µL of internal standard and 100 µL of β-glucuronidase from *E. Coli* for 60 minutes at 55°C. After hydrolysis, 100 µL of carbonate-bicarbonate buffer 1 M (pH 11) was added and the pH was adjusted to 9 with K_2_CO_3_ 0.1 M. Solid phase extraction was then carried out on a C18 cartridge (Bond Elut, 200 mg/3mL) with *tert*-butylmethylether as extraction solvent. The extracts were evaporated to dryness under a stream of nitrogen at 60°C. The dry extract was dissolved in 150 µL of mobile phase and 20 µL were injected into the liquid chromatography-mass spectrometry system.

*Liquid chromatography (LC)*

The LC analyses were performed using a binary gradient system and automatic injector. Reverse-phase liquid chromatography was done using an Agilent Zorbax SB-C8 column (2.1 × 100 mm, 1.8 μm particle size). The solvents were: 10 mM of ammonium formate /acetic acid (pH 4) (eluent A) and acetonitrile (eluent B).

After automatic injection the gradient program started at 30 % B for 1.5 min, then increased to 40 % B in 1.25 min, and then to 90 % B in 4.15 min. The column was re-equilibrated at 30 % B for 1 min. The flow rate was set at 440 μL.min^-1^ and the column temperature at 20 °C.

*Triple quadrupole MS/MS system*

We used a triple quadrupole system (Xevo, Waters) with positive and negative electrospray ionization using a desolvation gas flow of 800 L/hr, a cone gas flow of 50 L/hr, ion source temperature of 150°C, and desolvation temperature of 450°C. Selected reaction monitoring (SRM) was used for the acquisition mode (the ion transitions selected and the optimal collision energies are reported in S2 Table), with collision-induced dissociation (CID) using nitrogen as collision gas at 5.10^-3^ mBar.

- 1. **Screening procedure by LC-MS/MS without enzymatic hydrolysis**

*Sample pre-treatment*

To 2 mL of urine, 500 µL of acetate buffer 0.2 M (pH 5.2) and 40 µL of internal standard were added. A first liquid-liquid extraction was carried out with 2 mL of ethyl acetate. The organic layer was transferred to another tube and 500 µL of sodium hydroxide (98%) was added to the aqueous layer to bring it to pH 14. A second liquid-liquid extraction was then carried out with a further 2 mL ethyl acetate. The organic layer was pooled with the first organic layer. The extracts were evaporated to dryness under a stream of nitrogen at 60°C. The dry extract was dissolved in 150 µL of LC-MS/MS mobile phase and 20 µL were injected into the liquid chromatography-mass spectrometry system.

*Liquid chromatography*

The LC analyses were performed as described above except that the gradient program started at 10 % B, increased to 55 % B in 8 min, and then increased to 100 % B in 0.1 min. The column was re-equilibrated at 10 % B for 1 min.

*Triple quadrupole MS/MS system*

Tandem mass spectroscopy was performed as described above. The ion transitions selected and the optimal collision energies are reported in S3 Table.

- 1. **Screening procedure for anabolic steroids and metabolic modulators by gas chromatography (GC)-MS/MS**

*Sample pre-treatment*

2 mL of urine was hydrolyzed enzymatically as described above (section 1.1). The dry extracts were derivatized in MSTFA/NH4I/DTE (1000:2:3, v/w/w) (N-Methyl-N-trifluorotrimethylsilyl acetamide (MSTFA), ammonium iodide (NH_4_I), dithioerythritol (DTE)) for 20 min at 60°C. Then 1 µL was injected into the gas chromatography-mass spectrometry system.

*Gas chromatography*

The GC-MS/MS analyses were performed using an Agilent 7890A GC chromatograph with a 20 m fused-silica capillary column DB-5MS, i.d. 0.180 mm, film thickness 0.18 µm. The carrier gas was helium (constant flow, split ratio 1/20) and the temperature program was as follows: 120°C (hold 0.5 min), 15°C/min to 210°C, 5°C/min to 280°C, 40°C/min to 320 (hold 3.5 min). The temperature of the injection port was 280°C and the transfer line was set at 320°C.

*Triple quadrupole MS/MS system*

Analyses were performed using a triple quadrupole system 7000B from Agilent in electron ionization (70 eV) mode. The temperature of the quadrupole was set at 150°C and the temperature of the ion source was 230°C. Selected reaction monitoring (SRM) was used as the acquisition mode, with collision-induced dissociation (CID) using nitrogen as collision gas at 1.5mL/min.

- 1. **Confirmatory analyses**

A summary of the confirmatory procedures (i.e. sample preparation and type of analysis) used for each substance is presented in S4 Table.

1. **Analytical procedures for benzodiazepines (and related substances) and PDE-5 inhibitors**

The following analytical procedures are also reported in detail elsewhere [1].

*Sample pre-treatment*

2 mL of urine was incubated with 1 mL phosphate buffer (pH 7.4), 30 μL of β-glucuronidase from *E. coli* and 20 μL of internal standard for 60 minutes at 50 ºC. Following hydrolysis a liquid/liquid extraction was carried out with 7 mL of *tert*-butylmethylether for 5 minutes on a mechanical shaker; after centrifugation the organic phase was transferred and evaporated to dryness under a stream of nitrogen at 75 °C. The dried extract was dissolved in 50 µL of mobile phase and 10 µL were injected into the liquid chromatography-mass spectrometry system.

# *Liquid chromatography*

All LC analyses were performed using a binary gradient system and automatic injector. Reversed-phase liquid chromatography was on a Supelco Ascentis® C18 column (2.1 × 150 mm, 5 μm fused core particle). The solvents were: 0.1% formic acid and 5 mM ammonium formate (eluent A) and acetonitrile containing 0.1% formic acid (eluent B).

The gradient started at 10% B and increased to 60% B in 7 min; after 6 min at 60% B, it was increased to 100% B in 2 min. The column was flushed for 1 min at 100% B and finally re-equilibrated at 10% B for 5 min. The flow rate was set at 250 μL min^-1^ and the column temperature at 20 °C.

# *Triple quadrupole MS/MS system*

All experiments were performed using a triple quadrupole system with positive electrospray ionization using a curtain gas pressure of 25 psi, a ion source temperature of 500 °C, an ion source gas 1 pressure of 35 psi, an ion source gas 2 pressure of 40 psi, a declustering voltage of 80 V, an entrance potential of 10 V and a needle voltage of 5000 V. Selected reaction monitoring (SRM) was used as acquisition mode (the ion transitions selected and the optimal collision energies are reported in S5 Table), with collision-induced dissociation (CID) using nitrogen as collision gas at 5.8.10^-5^ mBar.

1. **Supplementary tables**

**S2 Table**. Mass spectrometric conditions (screening procedure with enzymatic hydrolysis).

|  |  |  |  |  |
| --- | --- | --- | --- | --- |
| **Compounds** | **Class** | **Retention Time** (min) | **Transition** (m/z) | **Collision energy** (eV) |
| Anastrozole | metabolic modulator | 10.7 | 209.2 > 115.1 | 5 |
| Betamethasone | corticosteroid | 3.30 | 393.2 > 355.2 | 11 |
| Budesonide | corticosteroid | 3.15 | 447.6 > 357.3 | 7 |
| Codeine | narcotic | 0.69 | 300.4 > 152.0 | 50 |
| Dihydrobupropion | stimulant | 1.74 | 242.1 > 167.9 | 19 |
| Hydrocodone | narcotic | 0.80 | 300.4 > 199.1 | 30 |
| Methoxytamoxifen | metabolic modulator | 5.20 | 418.3 > 331.3 | 30 |
| Metoprolol acid | beta-blocker | 0.64 | 268.3 > 145.0 | 23 |
| Morphine | narcotic | 0.59 | 286.4 > 181.2 | 37 |
| Prednisolone | corticosteroid | 2.45 | 361.3 > 343.4 | 9 |
| Prednisone | corticosteroid | 2.65 | 359.1 > 146.9 | 28 |
| THC | cannabinoid | 5.72 | 345.4 > 192.9 | 26 |
| Tramadol | narcotic | 1.15 | 264.3 > 246.2 | 10 |

Budesonide, 6βOH-Budesonide; Dihydrobupropion, Erythro Dihydrobupropion; Methoxytamoxifen, 3OH-4-Methoxytamoxifen; THC, tetrahydrocannabinol-m. Only compounds detected in urine (see Results, Table 1) are presented in S2 Table. Other screened compounds (not shown) were anabolic agents, beta-2 agonists, metabolic modulators, narcotics, cannabinoids and beta-blockers.

**S3 Table**. Mass spectrometric conditions (screening procedure without enzymatic hydrolysis).

|  |  |  |  |  |
| --- | --- | --- | --- | --- |
| **Compounds** | **Class** | **Retention Time** (min) | **Transition** (m/z) | **Collision energy** (eV) |
| Acetazolamide | diuretic | 1.75 | 221.0 > 82.8 | 15 |
| Benzoylecgonine | stimulant | 2.47 | 290.4 > 104.8 | 30 |
| Betaxolol | beta-blocker | 5.10 | 308.4 > 158.9 | 21 |
| Bisoprolol | beta-blocker | 4.23 | 326.5 > 115.8 | 17 |
| Caffeine | stimulant | 2.25 | 195.0 > 137.9 | 20 |
| Ephedrine/Pseudoephedrine | stimulant | 1.73 | 166.1 > 56.0 | 30 |
| Heptaminol | stimulant | 0.80 | 146.0 > 68.9 | 16 |
| Hydrochlorothiazide | diuretic | 2.40 | 296.1 > 269.0 | 18 |
| Methadone | narcotic | 6.60 | 278.4 > 234.2 | 30 |
| Metoprolol | beta-blocker | 3.38 | 268.2 > 148.1 | 23 |
| N-ethylnicotinamide | stimulant | 2.20 | 150.7 > 52.9 | 29 |

Methadone, methadone-m. Only compounds detected in urine (see Results, Table 1) are presented in S3 Table. Other screened compounds (not shown) were anabolic agents, beta-2 agonists, metabolic modulators, narcotics, cannabinoids and beta-blockers.

**S4 Table. Confirmatory analyses.**

| **Compounds** | **Sample preparation** | **Analysis** |
| --- | --- | --- |
| Acetazolamide, Methadone-metabolite | Liquid-liquid extraction with ethyl acetate (pH 7) | LC-MS/MS |
| Benzoylecgonine | Enzymatic hydrolysis (E. Coli) pH 6.5 Solid phase extraction with Bond Elut certify cartridges (elution Dichloromethane / Isopropanol (80/20) with 2% NH_4_OH) Derivatization with MSTFA/NH_4_I/DTE (1000:2:3, v/w/w) | GC-MS |
| Betaxolol, Metoprolol | Enzymatic hydrolysis (Helix Pomatia) Liquid-liquid extraction with diethylether (pH 9) Derivatization with methyl boronic acid | GC-MS |
| Bisoprolol | Solid phase extraction with reversed-phase/strong cation-exchange cartridges (elution Dichloromethane / Isopropanol (80/20) with 2% NH_4_OH) | LC-MS/MS |
| Heptaminol | Liquid-liquid extraction with diethyl ether (saturated with Na_2_SO_4_) (pH 14) | GC-MS |
| Hydrochlorothiazide | Liquid-liquid extraction with ethyl acetate (pH 7) (twice) | LC-MS/MS |
| 3OH-4-Methoxytamoxifen | Enzymatic hydrolysis (E. Coli) pH 6.5 Liquid-liquid extraction with tert-Butyl methyl ether (pH 9) | LC-MS/MS |

LC, liquid chromatography; MS, mass spectrometry; GC, gas chromatography.

**S5 Table. Mass spectrometric conditions for benzodiazepines and PDE-5 inhibitors.**

| **Compound** | **Retention Time**  (min) | **Q1**  (m/z) | **Q3**  (m/z) | **Collision energy**  (eV) |
| --- | --- | --- | --- | --- |
| **Benzodiazepines and related substances** | | | | |
| Alprazolam | 11.1 | 309 | 205, 381 | 40, 35 |
| hydroxylated Alprazolam * | - | 325 | 227, 279 | 40, 35 |
| Bromazepam | 9.6 | 316 | 182, 209 | 35, 35 |
| hydroxylated Bromazepam* | - | 332 | 286, 314 | 40, 35 |
| Brotizolam | 11.5 | 395 | 279, 314 | 40, 35 |
| Chlordiazepoxide | 8.4 | 300 | 227, 241 | 35, 25 |
| Clobazam | 11.9 | 302 | 225, 260 | 40, 35 |
| Clonazepam | 11.2 | 316 | 223, 270 | 40, 35 |
| 7-amino-Clonazepam* | - | 286 | 121, 222 | 40, 35 |
| Diazepam | 12.2 | 285 | 222, 257 | 40, 35 |
| Delorazepam | 11.8 | 305 | 242, 277 | 40, 40 |
| Etizolam | 11.5 | 343 | 289, 314 | 45, 35 |
| Flurazepam | 9.1 | 389 | 289, 316 | 45, 35 |
| Lorazepam | 11.1 | 321 | 275, 303 | 35, 25 |
| Lormetazepam | 12.0 | 335 | 288, 317 | 35, 25 |
| Midazolam | - | 326 | 244, 286, 291 | 35, 35, 40 |
| Nordiazepam | 11.1 | 271 | 243, 208 | 40, 35 |
| Oxazepam | 11.0 | 287 | 241, 269 | 35, 25 |
| Pinazepam | 13.3 | 310 | 241, 292 | 45, 35 |
| Triazolam | 11.5 | 343 | 279, 308, 315 | 40, 35, 35 |
| hydroxylated Triazolam* | - | 359 | 176, 331 | 45, 35 |
| Zaleplon | 10.6 | 306 | 236, 260 | 35, 25 |
| Zoplicone | 7.6 | 389 | 217, 245 | 40, 25 |
| Zolpidem | 8.1 | 308 | 235, 263 | 35, 30 |
| **PDE-5 inhibitors** | | | | |
| Sildenafil | 9.1 | 475 | 58, 100, 311 | 50, 45, 35 |
| hydroxylated Sildenafil* | 6.80 | 491 | 100 | 45 |
| demethylated Sildenafil* | 8.60 | 461 | 100 | 45 |
| Tadalafil | 11.4 | 390 | 268, 135 | 30, 35 |
| Vardenafil | 8.4 | 489 | 151, 72 | 35, 50 |
| deethylated Vardenafil * | 8.80 | 461 | 151 | 35 |

Q1, precursor ion; Q3, product ions; PDE-5, phosphodiesterase 5. *certified reference standards not available; reference data based on the information reported in the literature [2, 3]. All compounds searched in urine are presented in S5 Table.

**References**

1. Mazzarino M, Cesarei L, de la Torre X, Fiacco I, Robach P, Botre F. A multi-targeted liquid chromatography-mass spectrometry screening procedure for the detection in human urine of drugs non-prohibited in sport commonly used by the athletes. J Pharm Biomed Anal. 2016;117:47-60. doi: 10.1016/j.jpba.2015.08.007. PubMed PMID: 26342446.

2. Smink BE, Brandsma JE, Dijkhuizen A, Lusthof KJ, de Gier JJ, Egberts AC, et al. Quantitative analysis of 33 benzodiazepines, metabolites and benzodiazepine-like substances in whole blood by liquid chromatography-(tandem) mass spectrometry. Journal of chromatography B, Analytical technologies in the biomedical and life sciences. 2004;811(1):13-20. doi: 10.1016/j.jchromb.2004.03.079. PubMed PMID: 15458716.

3. Strano-Rossi S, Anzillotti L, de la Torre X, Botre F. A gas chromatography/mass spectrometry method for the determination of sildenafil, vardenafil and tadalafil and their metabolites in human urine. Rapid communications in mass spectrometry : RCM. 2010;24(11):1697-706. doi: 10.1002/rcm.4568. PubMed PMID: 20486268.
