# Supplementary material for: Drug Use on Mont Blanc: A Study Using Automated Urine Collection
Source: PLoS One. 2016 Jun 2;11(6):e0156786. doi: 10.1371/journal.pone.0156786 (PMC4890938; doi:10.1371/journal.pone.0156786)
Supplement: S1 Table — (DOCX) [file pone.0156786.s002.docx]

**S1 Table. Individual data of drug concentrations in urine samples from Mont Blanc climbers.**

| **Positive**  **sample** | **Hut** | **Substance 1** | **Concen-tration (ng/ml)** | **Substance 2** | **Concen-tration**  **(ng/ml)** | **Substance 3** | **Concen-tration (ng/ml)** | **Substance 4** | **Concen-tration**  **(ng/ml)** |
| --- | --- | --- | --- | --- | --- | --- | --- | --- | --- |
| #1 | COSM | Acetazolamide | [1885] |  |  |  |  |  |  |
| #2 | COSM | Acetazolamide | 389 |  |  |  |  |  |  |
| #3 | COSM | Acetazolamide | 6328 |  |  |  |  |  |  |
| #4 | COSM | Acetazolamide | 390 |  |  |  |  |  |  |
| #5 | COSM | Acetazolamide | 48 |  |  |  |  |  |  |
| #6 | COSM | Acetazolamide | 473 | Zopiclone | 739 |  |  |  |  |
| #7 | COSM | Acetazolamide | 217 |  |  |  |  |  |  |
| #8 | COSM | Acetazolamide | 12725 | Zolpidem | 0.9 | Bisoprolol | 64 |  |  |
| #9 | COSM | Acetazolamide | 716 |  |  |  |  |  |  |
| #10 | COSM | Acetazolamide | [507] | Prednisone | 776 | Prednisolone^§^ | 3823 |  |  |
| #11 | COSM | Acetazolamide | [100] |  |  |  |  |  |  |
| #12 | COSM | Acetazolamide | 207 |  |  |  |  |  |  |
| #13 | COSM | Acetazolamide | [780] | Hydrochlorothiazide | 812 |  |  |  |  |
| #14 | COSM | Acetazolamide | 112 |  |  |  |  |  |  |
| #15 | COSM | Acetazolamide | 362035 |  |  |  |  |  |  |
| #16 | COSM | Acetazolamide | 15520 |  |  |  |  |  |  |
| #17 | COSM | Acetazolamide | 13150 |  |  |  |  |  |  |
| #18 | COSM | Acetazolamide | 252325 | Zolpidem | 7.3 |  |  |  |  |
| #19 | COSM | Acetazolamide | 51265 |  |  |  |  |  |  |
| #20 | COSM | Acetazolamide | 23968 | Hydrochlorothiazide | 954 |  |  |  |  |
| #21 | COSM | Acetazolamide | 316400 | Zolpidem | 4.4 |  |  |  |  |
| #22 | COSM | Acetazolamide | 43 |  |  |  |  |  |  |
| #23 | COSM | Acetazolamide | 193 |  |  |  |  |  |  |
| #24 | COSM | Acetazolamide | [27] |  |  |  |  |  |  |
| #25 | COSM | Acetazolamide | [14] | Methylprednisolone | 1226 |  |  |  |  |
| #26 | GOUTER | Acetazolamide | 59 | Hydrochlorothiazide | 4 |  |  |  |  |
| #27 | GOUTER | Acetazolamide | 2760 |  |  |  |  |  |  |
| #28 | GOUTER | Acetazolamide | 76975 | Betamethasone | 13 |  |  |  |  |
| #29 | GOUTER | Acetazolamide | 14770 |  |  |  |  |  |  |
| #30 | GOUTER | Acetazolamide | [55] | Benzoylecgonine | [37] | Pseudoephedrine | 879 |  |  |
| #31 | GOUTER | Acetazolamide | 42650 | Zolpidem | 7.4 |  |  |  |  |
| #32 | GOUTER | Acetazolamide | [189] |  |  |  |  |  |  |
| #33 | GOUTER | Acetazolamide | 32940 |  |  |  |  |  |  |
| #34 | GOUTER | Acetazolamide | 77075 | Betamethasone | 42 |  |  |  |  |
| #35 | GOUTER | Acetazolamide | 215 |  |  |  |  |  |  |
| #36 | GOUTER | Acetazolamide | 67650 | Zolpidem | 0.1 |  |  |  |  |
| #37 | GOUTER | Acetazolamide | 3405 |  |  |  |  |  |  |
| #38 | GOUTER | Acetazolamide | 89425 | Metoprolol | 34 | Metoprolol acid^§^ | 17 |  |  |
| #39 | GOUTER | Acetazolamide | 8162 |  |  |  |  |  |  |
| #40 | GOUTER | Acetazolamide | 28300 |  |  |  |  |  |  |
| #41 | GOUTER | Acetazolamide | 10050 |  |  |  |  |  |  |
| #42 | GOUTER | Acetazolamide | [14] | Prednisone | 630 | Prednisolone^§^ | 600 |  |  |
| #43 | GOUTER | Acetazolamide | [16] |  |  |  |  |  |  |
| #44 | GOUTER | Acetazolamide | [27] |  |  |  |  |  |  |
| #45 | GOUTER | Acetazolamide | [70] |  |  |  |  |  |  |
| #46 | GOUTER | Acetazolamide | 62 | Zolpidem | 0.5 |  |  |  |  |
| #47 | GOUTER | Acetazolamide | 3436 | Betamethasone | 20 |  |  |  |  |
| #48 | GOUTER | Acetazolamide | 351 |  |  |  |  |  |  |
| #49 | GOUTER | Acetazolamide | 115 |  |  |  |  |  |  |
| #50 | GOUTER | Acetazolamide | [15] | Prednisone | 430 | Prednisolone^§^ | 390 |  |  |
| #51 | GOUTER | Acetazolamide | 53 |  |  |  |  |  |  |
| #52 | GOUTER | Acetazolamide | 20 |  |  |  |  |  |  |
| #53 | GOUTER | Acetazolamide | 491100 |  |  |  |  |  |  |
| #54 | GOUTER | Acetazolamide | 50488 |  |  |  |  |  |  |
| #55 | GOUTER | Acetazolamide | [75] |  |  |  |  |  |  |
| #56 | GOUTER | Acetazolamide | 56 |  |  |  |  |  |  |
| #57 | GOUTER | Acetazolamide | [15] |  |  |  |  |  |  |
| #58 | GOUTER | Acetazolamide | 6460 |  |  |  |  |  |  |
| #59 | GOUTER | Acetazolamide | [15] |  |  |  |  |  |  |
| #60 | GOUTER | Acetazolamide | [13] | Prednisone | 95 | Prednisolone^§^ | 157 |  |  |
| #61 | GOUTER | Acetazolamide | 15250 |  |  |  |  |  |  |
| #62 | GOUTER | Acetazolamide | 1904 | Zolpidem | 0.3 |  |  |  |  |
| #63 | GOUTER | Acetazolamide | [43] |  |  |  |  |  |  |
| #64 | GOUTER | Acetazolamide | 138 |  |  |  |  |  |  |
| #65 | GOUTER | Acetazolamide | 21213 |  |  |  |  |  |  |
| #66 | GOUTER | Acetazolamide | [51] |  |  |  |  |  |  |
| #67 | GOUTER | Acetazolamide | [34] |  |  |  |  |  |  |
| #68 | GOUTER | Acetazolamide | [535] |  |  |  |  |  |  |
| #69 | GOUTER | Acetazolamide | [123] |  |  |  |  |  |  |
| #70 | GOUTER | Acetazolamide | 127650 |  |  |  |  |  |  |
| #71 | GOUTER | Acetazolamide | [22] |  |  |  |  |  |  |
| #72 | GOUTER | Acetazolamide | 25380 |  |  |  |  |  |  |
| #73 | GOUTER | Acetazolamide | [11] |  |  |  |  |  |  |
| #74 | GOUTER | Acetazolamide | [14] | Hydrochlorothiazide | 95 |  |  |  |  |
| #75 | GOUTER | Acetazolamide | 50800 | Zolpidem | 0.2 |  |  |  |  |
| #76 | GOUTER | Acetazolamide | 44050 | Codeine | 40 | Morphine^§^ | 6 | Hydrocodone | 480 |
| #77 | GOUTER | Acetazolamide | 53 |  |  |  |  |  |  |
| #78 | GOUTER | Acetazolamide | 15884 |  |  |  |  |  |  |
| #79 | GOUTER | Hydrochlorothiazide | 1506 |  |  |  |  |  |  |
| #80 | GOUTER | Hydrochlorothiazide | 128 |  |  |  |  |  |  |
| #81 | GOUTER | Hydrochlorothiazide | 10048 | Benzoylecgonine | 439 | Prednisone | 590 | Prednisolone^§^ | 240 |
| #82 | COSM | Zolpidem | 0.3 |  |  |  |  |  |  |
| #83 | COSM | Zolpidem | 0.8 | THC | 14 |  |  |  |  |
| #84 | COSM | Zolpidem | 2.3 | Zaleplon | 3 |  |  |  |  |
| #85 | COSM | Zolpidem | 12.6 |  |  |  |  |  |  |
| #86 | COSM | Zolpidem | 17.2 |  |  |  |  |  |  |
| #87 | COSM | Zolpidem | 0.1 | Codeine | 133 | Morphine^§^ | 19 |  |  |
| #88 | COSM | Zolpidem | 1.1 |  |  |  |  |  |  |
| #89 | COSM | Zolpidem | 2.0 |  |  |  |  |  |  |
| #90 | COSM | Zolpidem | 8.8 |  |  |  |  |  |  |
| #91 | GOUTER | Zolpidem | 0.7 |  |  |  |  |  |  |
| #92 | GOUTER | Zolpidem | 9.7 | Prednisone | 580 | Prednisolone^§^ | 1356 |  |  |
| #93 | GOUTER | Zolpidem | 2.6 |  |  |  |  |  |  |
| #94 | GOUTER | Zolpidem | 4.8 | Caffeine | 8200 |  |  |  |  |
| #95 | GOUTER | Zolpidem | 21.5 |  |  |  |  |  |  |
| #96 | GOUTER | Zolpidem | 8.2 | Dihydrobupropion | 665 |  |  |  |  |
| #97 | GOUTER | Zolpidem | 0.4 |  |  |  |  |  |  |
| #98 | GOUTER | Zolpidem | 127.2 |  |  |  |  |  |  |
| #99 | GOUTER | Zolpidem | 0.1 |  |  |  |  |  |  |
| #100 | GOUTER | Zolpidem | 8.3 |  |  |  |  |  |  |
| #101 | GOUTER | Zolpidem | 9.5 |  |  |  |  |  |  |
| #102 | GOUTER | Zolpidem | 0.7 |  |  |  |  |  |  |
| #103 | GOUTER | Zolpidem | 2.9 |  |  |  |  |  |  |
| #104 | GOUTER | Zolpidem | 4.8 |  |  |  |  |  |  |
| #105 | COSM | Oxazepam | 36 |  |  |  |  |  |  |
| #106 | COSM | Oxazepam | 108 |  |  |  |  |  |  |
| #107 | GOUTER | Oxazepam | 15.9 |  |  |  |  |  |  |
| #108 | GOUTER | Oxazepam | 3.3 |  |  |  |  |  |  |
| #109 | GOUTER | Oxazepam | 18 |  |  |  |  |  |  |
| #110 | GOUTER | Oxazepam | 222 |  |  |  |  |  |  |
| #111 | COSM | Zopiclone | 114 |  |  |  |  |  |  |
| #112 | GOUTER | Zopiclone | 68 |  |  |  |  |  |  |
| #113 | GOUTER | Zopiclone | 1 |  |  |  |  |  |  |
| #114 | GOUTER | Zopiclone | 3 |  |  |  |  |  |  |
| #115 | GOUTER | Lorazepam | 160 |  |  |  |  |  |  |
| #116 | GOUTER | Bromazepam | 23 |  |  |  |  |  |  |
| #117 | COSM | Brotizolam | 1 |  |  |  |  |  |  |
| #118 | COSM | Prednisone | 16 | Prednisolone^§^ | 9 | THC | 14 |  |  |
| #119 | GOUTER | Prednisone | 11 |  |  |  |  |  |  |
| #120 | COSM | Budesonide | 19 |  |  |  |  |  |  |
| #121 | GOUTER | Budesonide | 11 |  |  |  |  |  |  |
| #122 | COSM | Methylprednisolone | 51 |  |  |  |  |  |  |
| #123 | COSM | Caffeine | 8300 |  |  |  |  |  |  |
| #124 | GOUTER | Caffeine | 7400 |  |  |  |  |  |  |
| #125 | GOUTER | Benzoylecgonine | 68 |  |  |  |  |  |  |
| #126 | GOUTER | Pseudoephedrine | 21000 |  |  |  |  |  |  |
| #127 | GOUTER | Ephedrine | 9 |  |  |  |  |  |  |
| #128 | GOUTER | N-ethylnicotinamide | 299 |  |  |  |  |  |  |
| #129 | COSM | Heptaminol | 127 |  |  |  |  |  |  |
| #130 | COSM | THC | 152 |  |  |  |  |  |  |
| #131 | COSM | THC | 48 |  |  |  |  |  |  |
| #132 | GOUTER | THC | 76 |  |  |  |  |  |  |
| #133 | GOUTER | THC | 15 |  |  |  |  |  |  |
| #134 | GOUTER | THC | 34 |  |  |  |  |  |  |
| #135 | GOUTER | THC | 18 |  |  |  |  |  |  |
| #136 | GOUTER | THC | 137 |  |  |  |  |  |  |
| #137 | GOUTER | THC | 17 |  |  |  |  |  |  |
| #138 | GOUTER | THC | 12 |  |  |  |  |  |  |
| #139 | GOUTER | THC | 62 |  |  |  |  |  |  |
| #140 | GOUTER | THC | 27 |  |  |  |  |  |  |
| #141 | GOUTER | THC | 21 |  |  |  |  |  |  |
| #142 | GOUTER | THC | 53 |  |  |  |  |  |  |
| #143 | GOUTER | THC | 8 |  |  |  |  |  |  |
| #144 | COSM | Codeine | 11 | Morphine^§^ | 73 |  |  |  |  |
| #145 | COSM | Codeine | 11 | Morphine^§^ | 274 |  |  |  |  |
| #146 | COSM | Codeine | 1297 | Morphine^§^ | 123 |  |  |  |  |
| #147 | GOUTER | Codeine | 50 | Morphine^§^ | 12 |  |  |  |  |
| #148 | GOUTER | Codeine | 720 | Morphine^§^ | 28 |  |  |  |  |
| #149 | GOUTER | Codeine | 778 | Morphine^§^ | 22 |  |  |  |  |
| #150 | COSM | Tramadol | 358 |  |  |  |  |  |  |
| #151 | GOUTER | Betaxolol | 212 |  |  |  |  |  |  |
| #152 | GOUTER | Betaxolol | 190 |  |  |  |  |  |  |
| #153 | GOUTER | Metoprolol | 54 | Metoprolol acid^§^ | 200 |  |  |  |  |
| #154 | GOUTER | Methoxytamoxifen | 25 | Anastrozole | 250 | Caffeine | 7000 | Lorazepam | 5556 |
|  |  | Methadone | 5 |  |  |  |  |  |  |

Urine concentrations are expressed in ng/ml. ^§^indicates that this substance was always detected concomitantly with the previous one, within the same urine sample. Only the first substance (i.e. prednisone, codeine or metoprolol) was recorded for positive cases, as well as for drug combination counting. For acetazolamide and benzoylecgonine, numbers in brackets report concentrations determined by screening analyses. Sample #154 indicates drug combination with five distinct substances.
